# Mortality risk for women on chronic hemodialysis differs by age

**DOI:** 10.1186/2054-3581-1-10

**Published:** 2014-06-03

**Authors:** Manish M Sood, Claudio Rigatto, Paul Komenda, Julie Mojica, Navdeep Tangri

**Affiliations:** Ottawa Hospital Research Institute, The Ottawa Hospital, Civic campus, 2-014 Administrative Services Building, 1053 Carling Avenue, Box 693, Ottawa, Ontario K1Y 4E9 Canada; Department of Medicine, Section of Nephrology, Seven Oaks Hospital, University of Manitoba, 2300 McPhillips Street, Winnipeg, R2V 3M3 Canada; Department of Medicine, Health Sciences Centre, University of Manitoba, 820 Sherbrook street, Winnipeg, Manitoba R3A 1R9 Canada

## Abstract

**Background:**

Previous reports have demonstrated similar survival for men and women on hemodialysis, despite women’s increased survival in the general population.

**Objectives:**

To examine the effect of age on mortality in women undergoing chronic hemodialysis.

**Design:**

A retrospective cohort study using an administrative data registry, the Canadian Organ Replacement Registry (CORR) from Jan. 2001 and Dec. 2009.

**Setting:**

Canada.

**Patients:**

28,971 (Women 11,792 (40.7%), Men 17,179 (59.3%)) incident chronic hemodialysis patients who survived greater than 90 days on dialysis.

**Measurements:**

All-cause mortality.

**Methods:**

Cox proportional hazards and competing risks models were employed to determine the independent association between sex, age and likelihood of all-cause mortality with renal transplantation as the competing outcome.

**Results:**

During the study period, 6060 (51.4%) of women and 8650 (50.4%) of men initiating dialysis died. Younger women experienced higher mortality (Age < 45: Women 22.5%, Men 18.2%, hazard ratio (HR) 1.31 (1.12-1.52)) whereas elderly women experience lower mortality (Age 75–85: Women 65%, Men 67.3%, HR 0.94 95% CI 0.88-0.99, Age > 85: Women 66%, Men 70.2%, HR 0.83 95% CI 0.71-0.97) compared to men. This relationship persisted after accounting for the competing risk of transplantation.

**Limitations:**

The cause of death was unknown.

**Conclusions:**

Women’s survival on chronic hemodialysis varies by age compared to men with a significantly higher mortality in women younger than 45 years old and lower mortality in woman older than 75 years of age.

## Abrégé

**Contexte:**

Des rapports précédents ont démontré une survie similaire pour les hommes et les femmes en hémodialyse, malgré une meilleure survie pour les femmes dans la population générale.

**Objectifs:**

Examiner l’incidence de l’âge sur la mortalité chez les femmes en hémodialyse chronique.

**Type d’étude:**

Une étude de cohorte rétrospective basée sur un registre de données administratives, le Registre canadien des insuffisances et des transplantations d’organes (RCITO), de janvier 2001 à décembre 2009.

**Participants:**

28 971 (femmes 11 792 (40,7%), hommes 17 179 (59,3%)) nouveaux patients souffrant d’insuffisance rénale et traités en hémodialyse, qui ont survécu plus de 90 jours.

**Mesures:**

Mortalité toutes causes confondues.

**Méthodes:**

On a eu recours au modèle des risques proportionnels de Cox et au modèle de probabilités concurrentes pour déterminer l’association indépendante entre le genre, l’âge et les probabilités de mortalité toutes causes confondues, et la transplantation rénale est considérée comme un résultat concurrent.

**Résultats:**

Au cours de la période d’étude, 6 060 (51,4%) des femmes et 8 650 (50,4%) des hommes qui ont entamé la dialyse sont décédés. La mortalité des jeunes femmes a été supérieure (âge < 45: femmes 22,5%, hommes 18,2%, rapport des risques (hazard ratio, HR) 1,31 (1,12-1,52)), alors que la mortalité des femmes âgées a été inférieure (âge 75-85: femmes 65%, hommes 67,3%, HR 0,9495% IC 0,88-0,99; âge >85: femmes 66%, hommes 70,2%, HR 0,83 95% IC 0,71-0,97) que celle des hommes. Ce rapport demeurait, après avoir pris en considération le risque concurrent de la transplantation.

**Limites de l’étude:**

La cause du décès est inconnue.

**Conclusions:**

La survie des femmes en hémodialyse chronique, comparativement à celle des hommes, varie selon l'âge. En effet, la mortalité des femmes de moins de 45 ans est significativement supérieure à celle des hommes du même âge alors que celle des femmes âgées de plus de 75 ans est inférieure à celle des hommes de plus de 75 ans.

## What was known before

Women in the general population experience increased longevity relative to men. Furthermore previous reports have suggested higher mortality in younger woman on hemodialysis. Whether this is true in a Canadian population, after accounting for multiple confounders and the competing risk of kidney transplantation, remains unknown.

## What this adds

We found that women’s survival on chronic hemodialysis varies by age with higher mortality in women younger than 45 years old and lower mortality in woman older than 75 years of age compared to men and this was persistent after accounting for the competing risk of transplantation.

## Background

Women comprise a significant and increasing proportion of the worldwide dialysis population. Several studies have identified significant differences in prevalence and treatment of kidney disease between the sexes. Women appear less likely than men to start dialysis for treatment of kidney failure and less likely to receive a kidney transplant [1–4]. Upon initiation of dialysis, women are more likely to receive a lower dialysis dose, inferior treatment of anemia and mineral metabolism complications, and have a catheter as a vascular access [5–7]. Although, attempts to mitigate sex-based differences in renal care have been attempted; significant disparities still exist [5].

Despite clearly documented differences in the delivery of dialysis care, the questions of whether these differences adversely impact the outcome of women on dialysis remains unclear. A small number of previous studies suggest younger women on hemodialysis may have a higher mortality relative to men [2, 8, 9]. Furthermore, elderly women have been reported to have comparable survival to elderly men on hemodialysis [2, 8, 9]. Whether these findings are applicable to the Canadian dialysis population remains unknown. In populations with disparate rates of censoring and competing events, such as differences in transplantation in end stage renal disease (ESRD), appropriate analyses must be employed to account for these differences [10, 11]. The use of competing outcomes analyses has allowed considerable insight into sex based disparities in transplantation rates on hemodialysis demonstrating elderly women are less likely to receive a renal transplant and young African Americans on hemodialysis experience a higher mortality [3, 12]. Relatively healthy elderly women may remain on hemodialysis, as opposed to healthy elderly men who may receive a transplant, creating a survival disparity. Accounting for the competing event of transplantation may alter the survival estimates of elderly women on hemodialysis such that it mimics observations from the general population, where women experience a survival benefit and improved longevity compared to men.

In this regard we set out to examine sex-based differences on mortality among a chronic hemodialysis cohort utilizing a competing risk analysis and whether age was an effect modifier. We hypothesized that women would experience higher survival after accounting for the competing risk of transplantation and this would differ by age.

## Results

### Sex and age-based patient characteristics

Women were the minority on hemodialysis therapies across all age categories (Table 1). They comprised a larger proportion of the hemodialysis population among Aboriginal and East Asian’s. Older women (>45 years of age) had a higher BMI and shorter pre-dialysis care than men. Older men (>45 years of age) had more cardiac and vascular disease, were more likely to be current cigarette smokers and have a higher number of co-morbid illnesses. Women were more likely to have interstitial disease as the etiology of ESRD, and had a lower serum albumin and hemoglobin. Younger women (<45 years or age) were more likely to have pulmonary edema and malignancy at dialysis initiation and less likely to be on an anti-hypertensive medication. Lastly women were significantly less likely to initiate hemodialysis with an arteriovenous fistula (AVF) or arteriovenous graft (AVG) across all age groups with absolute difference of 4.0 to 5.7%.

**Table 1:** **Characteristics of the study cohort stratified by sex and age categories**

Characteristic	Age < 45			Age 45-75			Age > 75		
	Women	Men	P value	Women	Men	P value	Women	Men	P value
**N %**	39.5(1406)	60.5(2152)		40.0(7044)	60.0(10585)		42.9(3342)	57.1(4442)	
**Age (±SD)**	35.5 ± 7.4	35.9 ± 7.2	0.1	63.4 ± 8.2	62.9 ± 8.3	<0.0001	81.0 ± 4.0	80.9 ± 4.0	0.5
**Race**			<0.0001			<0.0001			0.02
**Caucasian**	57.6(810)	65.1(1400)		70(4856)	74(7490)		77.8(2599)	80.6(3579)	
**Aboriginal**	15.9(223)	10.3(222)		7.3(644)	4.8(635)		1.6(53)	1.1(51)	
**East Asian**	6.8(95)	5.3(115)		5.1(359)	6.6(541)		7.8(260)	6.2(275)	
**Black**	6.1(86)	5.4(116)		3.4(724)	2.8(1234)		9.2(306)	8.9(397)	
**South Asian**	9.2(129)	9.3(201)		11.1(255)	9.1(345)		1.7(57)	1.3(58)	
**Other**	4.5(63)	4.6(98)		3.1(206)	2.7(340)		2.0(67)	1.8(82)	
**BMI (±SD)**	27.2 ± 8.6	26.9 ± 6.5	0.2	28.9 ± 8.1	27.9 ± 6.4	<0.0001	25.8 ± 6.1	25.4 ± 5.3	<0.0001
**Distance to nearest dialysis facility in km (IQR)**	11.4(5.0-62.2)	11.7(4.8-62.4)	0.7	12.0(4.9-59.5)	12.0(5.0-55.5)	0.9	8.6(4.0-28.8)	9.4(4.4-34.8)	0.06
**Any pre-dialysis care % (N)**	53.2(748)	50.9(1096)	0.2	58.5(4122)	58.7(6212)	0.8	57.2(1911)	59.2(2630)	0.07
**Median number of days with pre-dialysis care (IQR)**	562(220–1532)	574(211–1339)	0.9	133 (0–747)	140(0–812)	0.03	587 (217–1218)	666(246–1362)	0.003
**Geographic region % (N)**			0.08			0.06			0.2
**Atlantic**	9.2(129)	9.4(202)		11.6(815)	10.8(1137)		10.3(342)	10.4(461)	
**Central**	19.3(685)	31.6(1122)		51.6(3626)	53.2(5618)		59.2(1969)	57.7(2558)	
**Prairie**	12(425)	15.9(566)		24.3(1705)	23.2(2447)		17.6(586)	19.5(863)	
**Pacific**	11.8(166)	12.0(258)		12.5(882)	12.9(1367)		13(431)	12.5(555)	
**Co-morbidities: % (N)**									
**CAD**	5.1(72)	5.6(120)	0.6	22.3(1572)	23.7(2508)	0.04	26.4(881)	29.1(1292)	0.008
**AMI**	3.4(48)	3.8(82)	0.6	19.4(1370)	25.2(2666)	<0.0001	22.9(765)	30.3(1344)	<0.0001
**Pulmonary edema**	14.4(202)	11.6(249)	0.02	29.5(2080)	26.8(2837)	<0.0001	32(1070)	30.5(1357)	0.2
**DM**	35.2(495)	36(774)	0.7	56.6(3984)	55.1(5932)	0.06	38.3(1279)	36.1(1602)	0.05
**Stroke**	3.8(54)	3.3(70)	0.4	14.0(988)	14.6(1541)	0.3	16.7(557)	19.5(865)	0.002
**PVD**	6.7(94)	6.7(144)	0.9	17.9(1263)	22.4(2370)	<0.0001	17.7(590)	23.8(1055)	<0.0001
**Malignancy**	3.4(48)	1.8(38)	0.002	10.7(757)	11.5(1217)	0.2	15(500)	21.5(957)	<0.0001
**Lung disease**	3.4(48)	2.3(49)	0.05	12.7(894)	11.5(1215)	0.02	12.1(405)	15.4(683)	<0.0001
**HTN**	71.3(1003)	77.6(1670)	<0.0001	83.3(5870)	83.2(8803)	0.8	84.4(2819)	82(3642)	0.006
**Serious illness**	9.7(136)	8.5(183)	0.3	11.3(794)	11.3(1198)	0.9	9.4(314)	9.8(437)	0.5
**Current smoker**	18.5(260)	22.6(487)	0.003	13.4(943)	15.8(1675)	<0.0001	5.3(176)	7(313)	0.001
**CABG**	2.6(36)	1.9(41)	0.2	10.6(748)	16.9(1794)	<0.0001	12.3(412)	20.5(909)	<0.0001
**Number of co-morbidities (±SD)**	1.8 ± 1.4	1.8 ± 1.3	0.4	3.0 ± 1.9	3.2 ± 1.9	<0.0001	2.9 ± 1.8	3.3 ± (1.9	<0.0001
**Cause of ESRD % (N)**			0.02			<0.0001			<0.0001
**Vascular**	6.9(97)	9.3(200)		17.2(1214)	18.3(1938)		39.3(1312)	39.3(1744)	
**DM**	31.1(437)	31.4(676)		45.8(3227)	44.3(4694)		26.8(897)	25(1111)	
**GN**	33.6(472)	31.9(687)		12.2(859)	13.7(1449)		8.7(291)	8.8(389)	
**Obstruction**	4.6(64)	3.9(83)		2.2(153)	3.0(314)		2.4(81)	4.0(179)	
**Interstitial**	1.6(22)	0.6(13)		1.3(94)	0.7(74)		1.6(53)	0.9(38)	
**PCKD**	5.8(82)	5.3(114)		4.6(323)	4.2(445)		1.8(61)	1.6(73)	
**Other**	7.8(109)	8.2(177)		8.6(607)	7.9(841)		6.5(217)	6.3(279)	
**Unknown**	8.7(123)	9.4(202)		8.0(567)	7.8(830)		12.9(430)	14.2(629)	
**Serum Albumin g/L (±SD)**	30.1 ± 10.5	31.5 ± 10.3	<0.0001	31.1 ± 9.5	31.8 ± 9.3	<0.0001	32.2 ± 8.8	32.2 ± 7.1	0.8
**Hemoglobin g/L (±SD)**	93.9 ± 26.7	97.1 ± 23.9	<0.0001	99.5 ± 32.2	100.3 ± 21.1	0.03	102.0 ± 25.3	102.7 ± 23.9	0.2
**AVF/AVG % (N)**	11.6(163)	17.3(372)	<0.0001	16.6(1168)	20.6(2181)	<0.0001	14.5(483)	18.9(838)	<0.0001

N number, % percentage, SD standard deviation, BMI body mass index, IQR interquartile range, km kilometer, Atlantic include Newfoundland, New Brunswick, Nova Scotia, prince Edward Island, Central Ontario, Prairie Manitoba, Saskatchewan, Alberta, Pacific British Columbia. CAD coronary artery disease, AMI acute myocardial infarction, DM diabetes mellitus, HTN hypertension, CABG coronary artery bypass graft, ESRD end stage renal disease, g/L grams per litre, Any pre-dialysis was defined as contact with a Nephrologist 30 days prior to renal replacement therapy initiation, AVF/AVG arteriovenous fistula/arteriovenous graft.

### Association of sex, age and mortality

**Figure 1:**
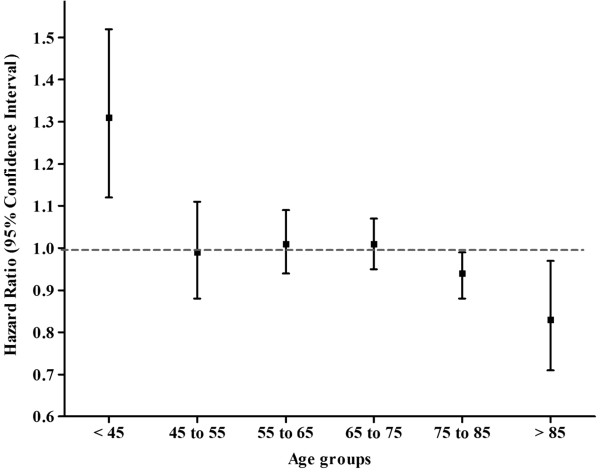
**Adjusted survival in women on hemodialysis stratified by age with men as the referent.** *Model adjusted for demographics, co-morbidities, body mass index (BMI), distance from centre, pre-dialysis care, cause of end stage renal disease, geographic region, serum hemoglobin and albumin.

In both unadjusted and adjusted models, mortality for women was similar to men (HR 0.99 95% CI 0.96-1.03) however it varied by age groups (age X sex interaction p = 0.002). In individuals < 45 years of age, there were 317 (22.5%) and 391 (18.2%) deaths in women and men respectively, an absolute difference of 4.3% (crude mortality rate: women 4.53 and men 3.49 per 100 patient years, Table 2). After adjustment, this difference in mortality persisted as women under the age of 45 had a higher mortality compared to men (HR 1.31 95% CI 1.12-1.52) (see Figure 1). Conversely, women over 75 years of age experienced a modest survival benefit relative to men (age 75–85: HR 0.93 95% CI 0.87-0.99, age > 85: HR 0.82 95%CI 0.70-0.96).

**Table 2:** **Crude numbers and proportions of mortality on hemodialysis in women according to age categories with men as the referent**

Age group (in years)	Women		Male	
	Number of deaths	Proportion of total	Number of deaths	Proportion of total
**<45**	317	22.5	391	18.2
**45-55**	499	35.0	833	35.2
**55-65**	1120	48.3	1667	46.6
**65-75**	1947	59.0	2753	59.3
**75-85**	1880	65.0	2568	67.3
**>85**	297	66.0	438	70.2

### Association of sex, age and mortality accounting for transplantation

In our analyses accounting for transplantation we used fewer age categories (<40, 40–50, 51–60, >60) to reflect that few individuals greater than 60 years of age receive a renal transplant. Utilizing a Fine and Gray model accounting for the competing risk (hazard ratio competing HRc) of transplantation, the hazard ratio for mortality in women compared to men was similar as traditional cox models (HR) (age < 40: HRc 1.32 (1.14-1.54) HR 1.35 (1.10-1.66), age 40–50 HRc 1.01 (0.90-1.13) HR 1.04 (0.90-1.20), age 51–60 HRc 1.03 (0.95-1.11) HR 1.01 (0.93-1.11), age > 60 HRc 1.02 (0.96-1.08) HR 1.00 (0.96-1.04). Women were less likely to receive a renal transplant compared to men, especially in those > 50 years of age (age 51–60: HRc 0.65 95% CI 0.55-0.77, > 60: HRc 0.56 95% CI 0.41-0.76).

## Discussion

In this large national cohort of patients on chronic hemodialysis, substantial mortality differences were evident among the sexes based on age. Although overall mortality for men and women after comprehensive adjustment was similar, age was a significant effect modifier as women under the age of 45 years of age had a substantially higher mortality risk compared to men of a similar age. In contrast, women over the age of 75 years of age experienced a modestly lower mortality, an observation consistent with data in the general population. These differences persisted after accounting for the competing risk of transplantation and after comprehensive adjustment for confounders.

The finding that mortality is increased in women < 45 years of age on hemodialysis relative to men is congruent with finding from previous studies from Europe and the United States [2, 8, 9]. Crude mortality rates for hemodialysis patients from the United States Renal Data System (USRDS) show higher mortality in women under the age of 60 years of age compared to men with the largest disparity among those aged 20–29 years of age (Crude mortality rate male 4.93 women 6.29 per 100 patient years; relative rate 1.27). Similarly, data from the Renal Epidemiology and Information Network (REIN) registry in France reported an increase in the standardized mortality ratio in women relative to men, predominantly due to mortality in the first four years on dialysis. Our findings are consistent with these previous reports and demonstrate the mortality difference persists after adjustment for numerous confounders and after accounting for the competing outcome of transplantation.

We observed women greater than 75 years of age had a higher mortality compared to men. We found absolute mortality reductions of 2.3% and 4.2% in women 75 to 85 years of age and over 85 years of age relative to similar aged men. This observation mimics those seen in the Canadian general population where women experience, on average, an additional 4 years of longevity relative to men [13]. However it is unclear if the survival benefit is simply an extension of those observed in the general population or due to the possibility of a selection bias. There have been numerous reports that elderly women do not receive equal access to cardiovascular procedures, surgical interventions and kidney transplantation [14–16]. Elderly women may not choose or be offered chronic hemodialysis to the same extent as men thus altering the population on chronic hemodialysis. Further investigations regarding sex-based differences in acceptance and offer of dialysis treatment are required.

It is well known that kidney transplantation as a treatment for kidney failure is less frequent among women than men. In our cohort, we also found that the likelihood of receiving a kidney transplant was lower across all age categories in women. However, these differences do not explain the age-related mortality differences among the sexes since, after accounting for the competing risk of transplantation, there was little change in the observed mortality risk. Thus young women on dialysis are disadvantaged by both a higher risk of mortality and a reduced likelihood of receiving a renal transplant.

We did find several risk factors that may contribute to the higher mortality observed in younger woman on dialysis. Woman less than 45 years of age had an almost two-fold increase in malignancy, were more likely to have glomerulonephritis, obstruction or interstitial disease as their cause of renal failure, have a lower serum albumin and hemoglobin and were 5.7% less likely to initiate dialysis with a fistula. Of these only vascular access represents a potentially modifiable risk factor. Multiple studies have reported lower rates of AVF among women however it remains unclear if this represents actual differences in patient agreement for AVF, referral rates, AVF creation and/or maturation among the sexes [17–19]. Technical difficulties may include smaller vessel diameter or early thrombosis. Miller *et al.* reported post-AVF creation women are less likely to achieve fistula patency and are more likely to have early thrombosis and undergo more salvage procedures compared to men. A similar increase in complications and the need for more revascularization procedures has been reported in AVG in women compared to men [20]. It is possible that there may be important cosmetic considerations as young women may be less agreeable to AVF surgery. Attitudes towards vascular access suggest body image is an important consideration however whether it is sex-specific remains unclear [21].

The main strengths of our study are the use of a large national cohort with over 10 years of incident patient follow up. CORR captures data on almost all patients who initiate hemodialysis in Canada. We were able to perform more comprehensive covariate adjustment than previous studies including co-morbidities, modality, vascular access, laboratory parameters, geography, pre-dialysis care and distance to dialysis facility. We also accounted for the competing outcome of transplantation in our survival analysis.

Conversely, our study had limitations. As in any observational study, residual confounding cannot be ruled out as an alternative explanation for our findings. We lacked detailed information regarding the cause of death. We did not account for modality transitions namely initiation of dialysis on peritoneal and transitions to hemodialysis. Our vascular access measurements were taken at initiation of dialysis, did not reflect changes that occur over time and may not reflect access function. The distance from dialysis centre was direct linear distance and may not accurately reflect driving distances or travelling time. We lacked information on the source (living, deceased) of organ procurement.

## Conclusion

In conclusion we found that age is an important effect modifier in survival of women on hemodialysis. Young women experienced a considerably higher mortality relative to men whereas older women appear to have a survival advantage. This was consistent after accounting for the competing risks of renal transplantation. Further sex-specific investigations are required into the mechanisms and possible prevention of these mortality disparities.

## Methods

### Study design and cohort development

This study was approved by the Research Board and the Hospital Ethics Board at St. Boniface Hospital in Winnipeg, Manitoba. We used the Canadian Organ Replacement Registry (CORR) as the source cohort for this analysis. CORR is a validated ESRD registry that captures data on all dialysis patients in Canada including demographics, death, dialysis modality, comorbidities and transplantation who supply written consent [22, 23]. We included all adult patients starting hemodialysis between January 1, 2001 and December 2009 who survived greater than 90 days. Patients with missing co-morbidity information were excluded (N =1,408). The final analytic cohort included 28,971 (Women 11,792 (40.7%), Men 17,179 (59.3%)). All data was de-identified, retrospective from the CORR and thus individual participant consent was not required.

### Definitions

Co-morbid illnesses included a history of coronary artery disease, acute myocardial infarction, diabetes mellitus, pulmonary edema, peripheral vascular disease, malignancy, hypertension medication usage, current cigarette smoker, lung disease, any serious illness and stroke. Serious illness was defined as any illness that could shorten life expectancy to less than 5 years. Causes of ESRD included vascular disease, diabetes mellitus, glomerulonephritis, interstitial disease, polycystic kidney disease, obstruction, other and unknown. Provinces and territories were categorized as geographic regions as follows: Atlantic (New Brunswick, Nova Scotia, Prince Edward Island, Newfoundland), Central (Ontario), Prairies (Alberta, Saskatchewan, Manitoba, Nunavut, Northwest Territories), Pacific (British Columbia, Yukon). Any pre-dialysis care was defined as contact with a Nephrologist for 30 or more days prior to hemodialysis initiation. Distance to centre was calculated as the direct linear distance in kilometres between a patients postal code from their primary residence at dialysis initiation to the nearest dialysis provider. Comorbidities and laboratory data were ascertained at the onset of ESRD. The outcome of interest was all-cause mortality and follow-up for outcomes was until December 31^st^, 2009.

### Statistical analyses

Continuous variables of interest were summarized as mean or medians with standard deviation or inter-quartile range as appropriate. Differences in baseline characteristics were determined by student’s t-test for continuous variables and chi-square or the Mann–Whitney test for dichotomous variables.

To assess the relationship between the sexes and mortality, we examined both traditional cause-specific cox proportional hazards model and the modified risks regression model of Fine and Gray to account for the competing risk of transplantation [24]. Models were adjusted for demographics, co-morbidities, body mass index (BMI), distance from centre, pre-dialysis care, cause of ESRD, geographic region, serum hemoglobin and albumin. The competing risk accounted for in the Fine and Gray models was renal transplantation.

To assess whether age was an effect modifier for mortality among the sexes, an age X sex interaction term was added separately to the traditional cox and competing risks models. In separate models, age was entered both as a continuous variable and categorical variable (age < 45, 45–55, 55–65, 65–75, 75–85, 85+ years). Categories were created based on the number of death events and individual models were created per age stratum with men as the referent. As there are few kidney transplants with increasing age, age categories were collapsed in the competing risk models.

Multiple imputation was employed for missing values with a random draw from the predictive distribution from an imputation model repeated ten times [25]. The proportion and covariates with missing data that was imputed included BMI (8.2%), distance from centre (2.0%), cause of ESRD (2.9%), geographic region (0.2%), serum hemoglobin (15.4%) and albumin (21.5%). An iterative Markov chain Monte Carlo (MCMC) method was used and pooled estimates of 10 rounds of imputation reported. Imputed and non-imputed models were compared and as there were no substantive changes in point estimates, the pooled imputed results of 10 rounds of imputation were reported. Analyses were performed using PASW Version 18 and the Fine and Gray analyses were performed using R. All hypothesis tests were two sided with statistical significance to find as having a P value of <0.05.
